# Digital Ischemia and Gangrene: An Unusual Presentation of Chronic Myeloid Leukemia

**DOI:** 10.7759/cureus.70426

**Published:** 2024-09-29

**Authors:** Vijaya L Patil, Deepak R Chavan, Yashaswini Thimmarayappa, Linette P Mathias

**Affiliations:** 1 General Surgery, Shri B. M. Patil Medical College, Hospital and Research Centre, Vijayapura, IND

**Keywords:** chronic myeloid leukemia (cml), digital ischemia, digit gangrene, hyperviscosity, leukostasis

## Abstract

Digital ischemia with gangrene is a rare manifestation of chronic myeloid leukemia (CML). We report a rare CML case with severe thrombocytosis presenting clinically as digital ischemia and gangrene, the sole primary symptom. A 40-year-old patient presented with pain and discoloration of the tips of the ring and little fingers of the right hand for 20 days, which later progressed to dry gangrene with a line of demarcation and pre-gangrenous changes in the index and middle fingers. He was a chronic smoker and alcoholic with no other comorbidities. On investigation, he was found to have abnormal leukocytosis (153,000/mm^3^) and moderate thrombocytosis (969,000/mm^3^) with anemia. In view of abnormal leukocytosis, he was investigated for leukemia. He was found to be positive for BCR-ABL by reverse transcription polymerase chain reaction (RT-PCR), thus confirming the diagnosis of CML. He received imatinib 400 mg/day and subsequently experienced resolution of symptoms and complete hematological response by the 12th week of therapy.

## Introduction

Digital ischemia is a type of acral vascular syndrome that may occur due to various factors such as Buerger's disease, thromboembolism, trauma, smoking, autoimmune connective tissue disorders, vasculitis, substance abuse, etc. Digital ischemia may present as paraneoplastic syndrome in certain malignancies such as adenocarcinomas of the lung, breast, and digestive system and hematologic and gynecological malignancy [1]. Myeloproliferative disorders such as chronic myeloid leukemia (CML) with thrombocythemia presenting with digital ischemia and gangrene are extremely rare. Till now, only three cases have been reported in the literature as per our knowledge [1,2]. The exact mechanism for such a phenomenon is not well described in the literature. Leukostatic symptoms due to hyperviscosity in CML could be one of the causes. Leukostatic symptoms due to blood hyperviscosity may include headaches, dizziness, altered sensorium, blurred vision, cerebrovascular accidents, priapism, and digital gangrene. Leukostatic symptoms may be due to the production of cytokines and adhesion molecules by circulating blast cells, thus blocking microvasculature [3-6]. Thus, it poses a medical emergency and responds very well to leukapheresis and less rapidly to chemotherapy [3]. Recently, it has been proved that cytokine inhibitors and adhesion molecule antagonists have promising effects in preventing these symptoms. Imatinib therapy has shown excellent results in patients with chronic-phase CML, with complete cytogenetic remission seen in 76.2% and improvement in long-term survival in 89% [7-9]. This report presents an unusual primary presentation of a CML patient with digit gangrene managed with chemotherapy and heparin with an excellent complete clinical and hematological response.

## Case presentation

A male patient in their 40s was admitted with pain and discoloration of the tips of the ring and little fingers of the right hand for 20 days. Later, it progressed to digital gangrene of the distal phalanx with a line of demarcation within a period of one week. He also noticed pre-gangrenous changes in the tips of the index and middle fingers. There was no history of abdominal swellings, fatigue, anorexia, weight loss, jaundice, or swellings elsewhere on the body. He denied a history of trauma, drug abuse, addictions, and local interventions to the upper limbs. He was not a known diabetic or hypertensive patient and was not on any chronic medication. His profession involved washing utensils for long periods. He gave a history of ethanol consumption and tobacco consumption over 20 years.

**Figure 1:**
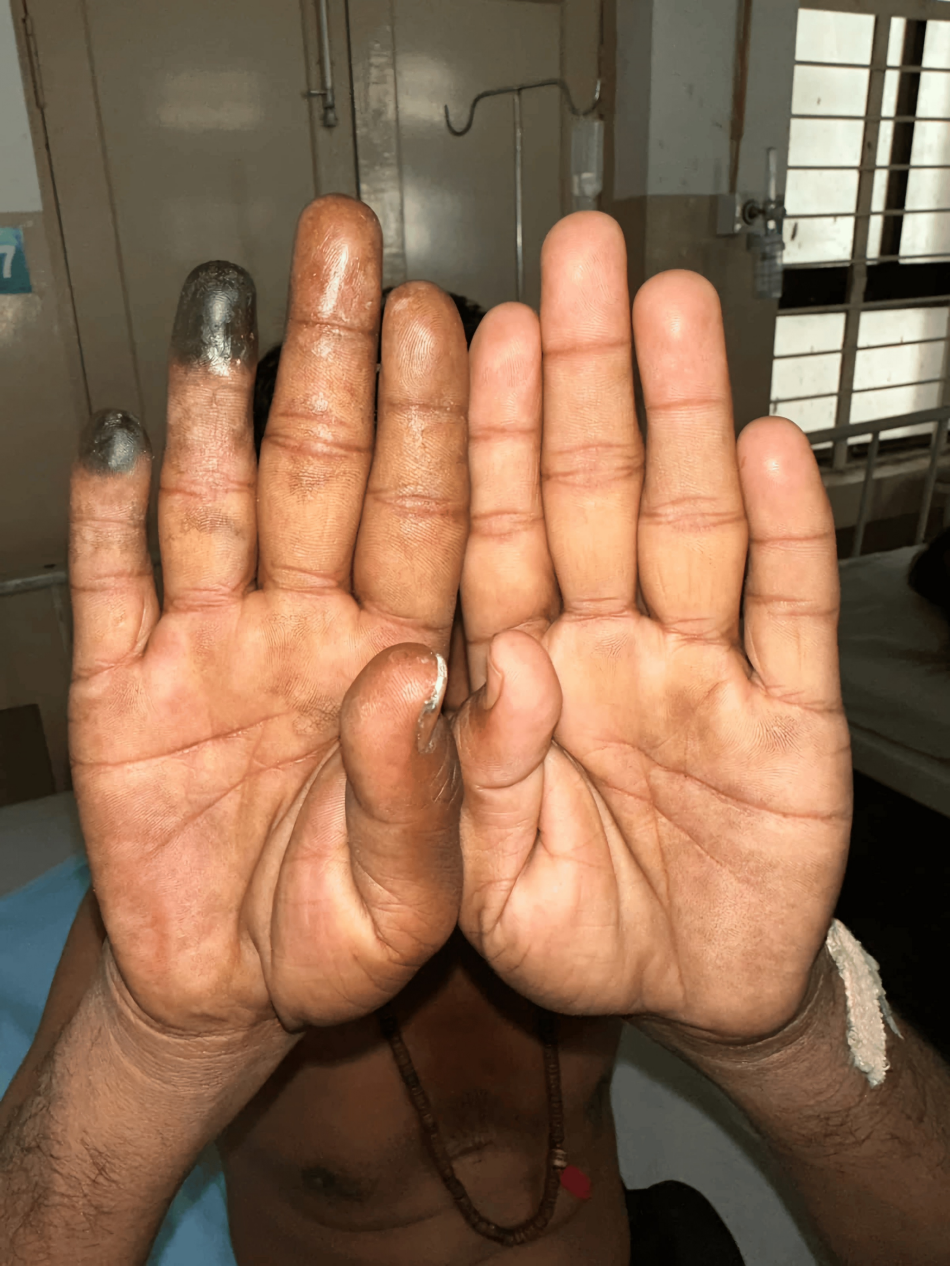
Digital gangrene and its progression in two weeks

Examination revealed a middle-aged man with mucosal pallor, with no icterus and enlarged lymph nodes. Right upper limb examination revealed dry gangrene of the distal phalanx of the ring and little fingers of the right hand with a line of demarcation, and the pre-gangrenous changes with tenderness were noted over the index and middle fingers (Figure 1). All the peripheral pulsations were normal. Their blood pressure was 130/80 mmHg.

Complete blood count (CBC) showed hemoglobin of 6 g/dl (normocytic normochromic), an increased total leukocyte count of 153.96×103/µL (54% neutrophils, 8% lymphocytes, 13% monocytes, 1% blasts, 2% promyelocytes, 7% metamyelocytes, and 11% band forms), and an increase in platelet count of 969x103/µL (Table 1).

**Table 1:** Laboratory investigations BCR: breakpoint cluster region; ABL: Abelson proto-oncogene

Parameters	Patient values	Reference range
Complete blood count		
Hemoglobin	6 g/dL	13-17 g/dL
Packed cell volume	25.8%	36-46%
Red blood count	2.99 million/cumm	3.8-4.8 million/cumm
Mean corpuscular volume	86 fL	83-101 fL
Mean corpuscular hemoglobin	20.06 pg	27-32 pg
Mean corpuscular hemoglobin concentration	23.25 g/dL	32-35 g/dL
Whole blood count	153.96 10^3^/µL	4-10 10^3^/µL
Neutrophils	54%	40-80%
Lymphocytes	8%	20-40%
Eosinophils	1%	1-6%
Monocytes	13%	2-10%
Basophils	0%	0-2%
Platelet count	969 10^3^/µL	150-410 10^3^/µL
Peripheral smear: differential leukocyte count		
Blasts	1%	
Promyelocytes	0%	
Myelocytes	3%	
Metamyelocytes	7%	
Band forms	11%	
Neutrophils	54%	
Lymphocytes	8%	
Monocytes	13%	
Eosinophils	1%	
Basophils	0%	
Bone marrow study: differential count		
Erythroblasts	13%	13.6-38.2%
Blasts	4%	0-2%
Promyelocytes	16%	2-4%
Myelocytes	24%	8-16%
Metamyelocytes	18%	10-25%
Band forms	12%	9-18%
Neutrophils	9%	7-14%
Eosinophil precursors	4%	1-4%
Basophils	0%	0-0.2%
Myeloid-to-erythroid ratio	6.15:1	1.5-3.3
Perls stain score	1	
Abnormal cells	Blasts: 4%	
Cytogenetic study		
BCR/ABL (MAJOR p210) copy number	9581.5 (positive)	
International scale normalized copy number	37.385%	0.003%

**Figure 2:**
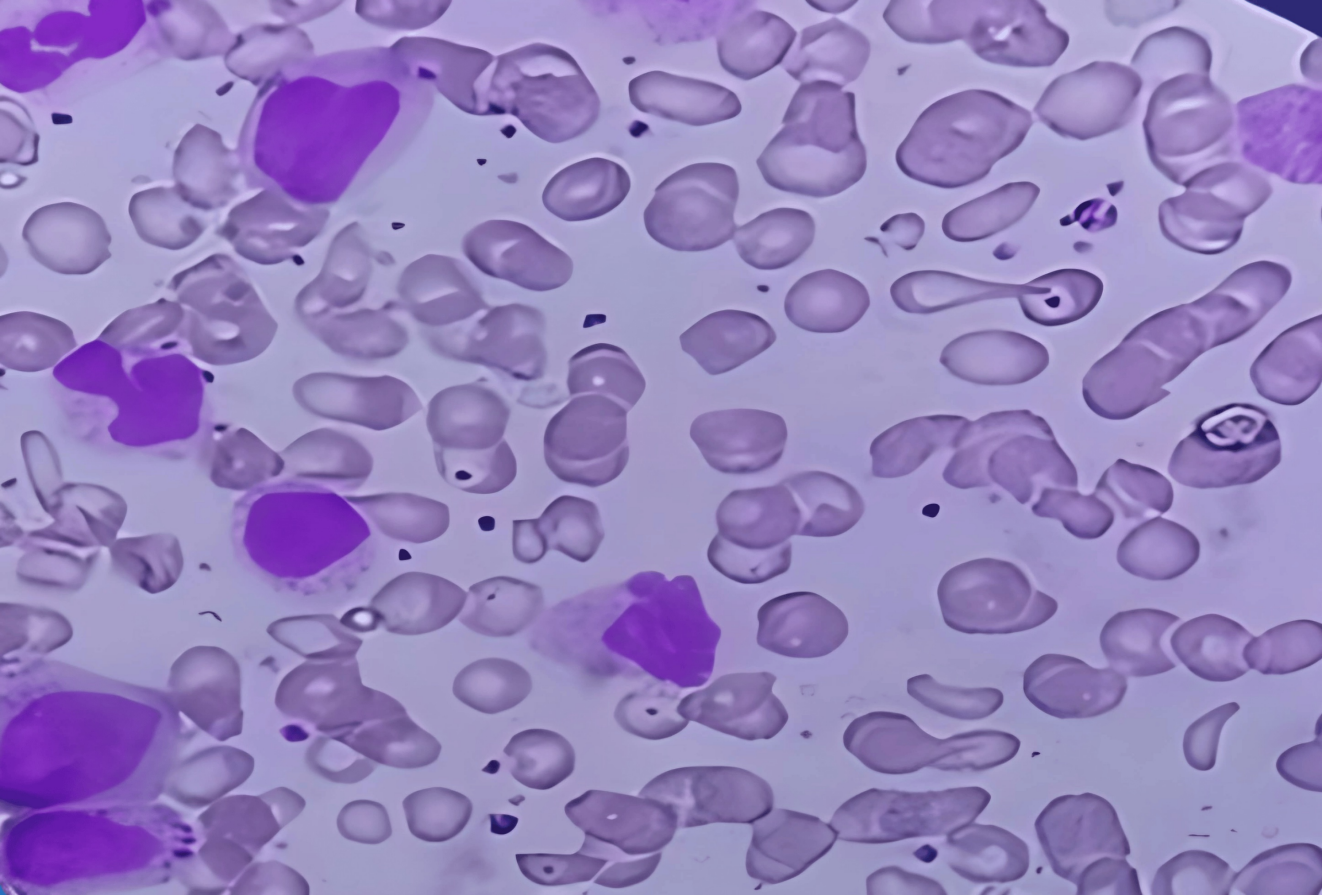
Microscopic image of the peripheral smear (Leishman stain, oil immersion): blast cells, thrombocytosis, and eosinophilia

**Figure 3:**
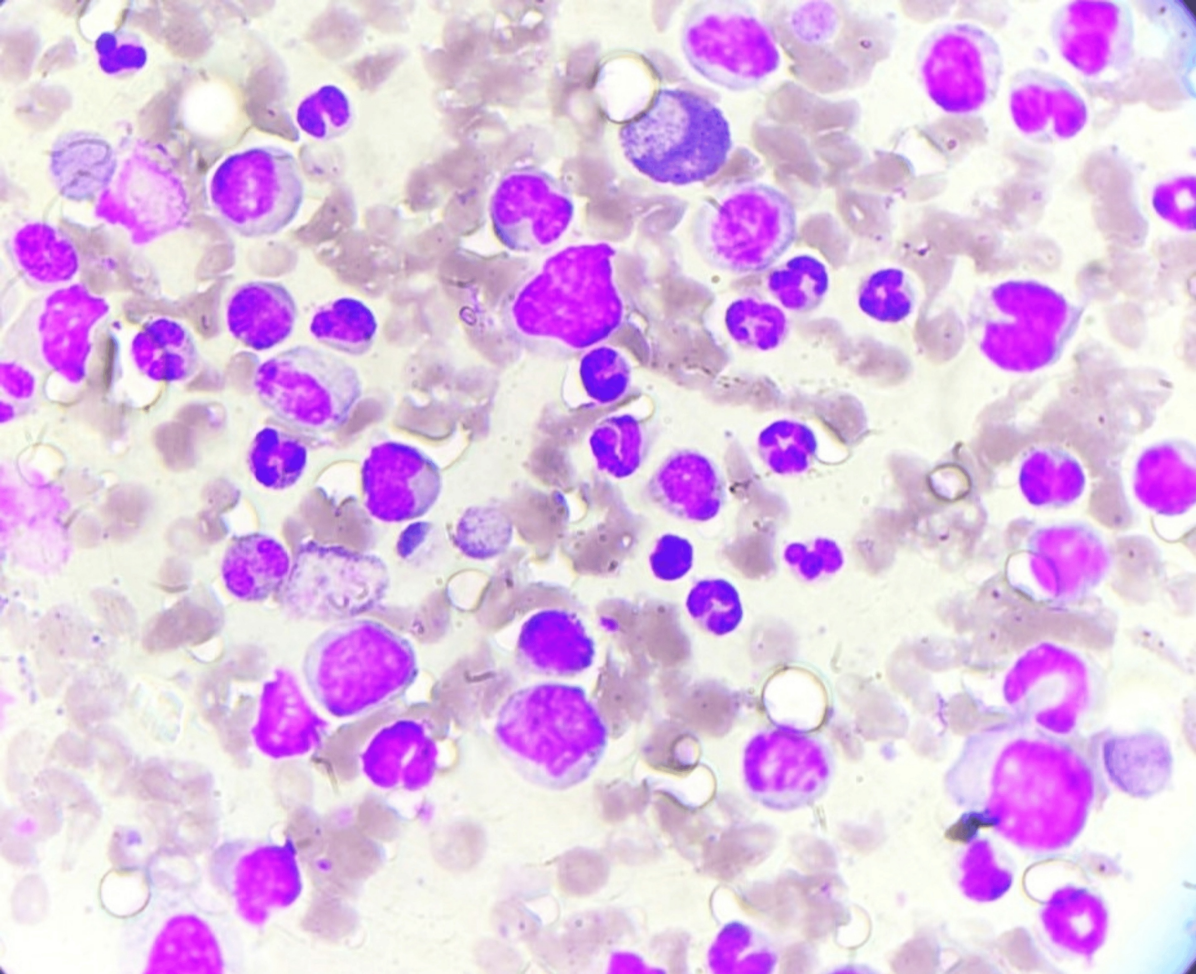
Bone marrow aspirate (Giemsa stain, 40×): myeloid hyperplasia with band forms, blast cells, promyelocytes, and metamyelocytes

Peripheral blood smear, differential counts (Figure 2), and bone marrow aspirates diagnosed the case as chronic-phase CML (Figure 3).

A biochemical study revealed normal blood sugar and serum electrolyte levels. Renal function tests and liver function tests were normal. Arterial color Doppler of both upper limbs was normal. Chest X-ray, electrocardiogram (ECG), and echocardiography were normal. Ultrasonography of the abdomen revealed mild hepatosplenomegaly. Cytogenetic study reverse transcription polymerase chain reaction (RT-PCR) for BCR-ABL (Philadelphia chromosome) was positive, thus confirming CML (Table 1).

Autoimmune disorders and hematological malignancy are to be kept in mind as the causes of digital gangrene after ruling out the source of thromboembolism: trauma. In view of abnormal leukocytosis and moderate thrombocytosis, the possibility of myeloproliferative disorders, essential thrombocytosis, and CML was considered. However, the diagnosis of CML was confirmed by a positive BCR-ABL gene.

While awaiting reports of BCR-ABL, in view of abnormal leukocytosis and thrombocytosis revealed by peripheral study and bone marrow aspirate, the patient was started on therapy hydroxyurea 500 mg (2 g daily in divided doses) with injection low-molecular-weight heparin (40 mg daily). On the availability of the report of BCR-ABL on the 10th day, he commenced on tyrosine kinase inhibitor tablet imatinib 400 mg and tablet Ecosprin and discontinued hydroxyurea therapy.

**Figure 4:**
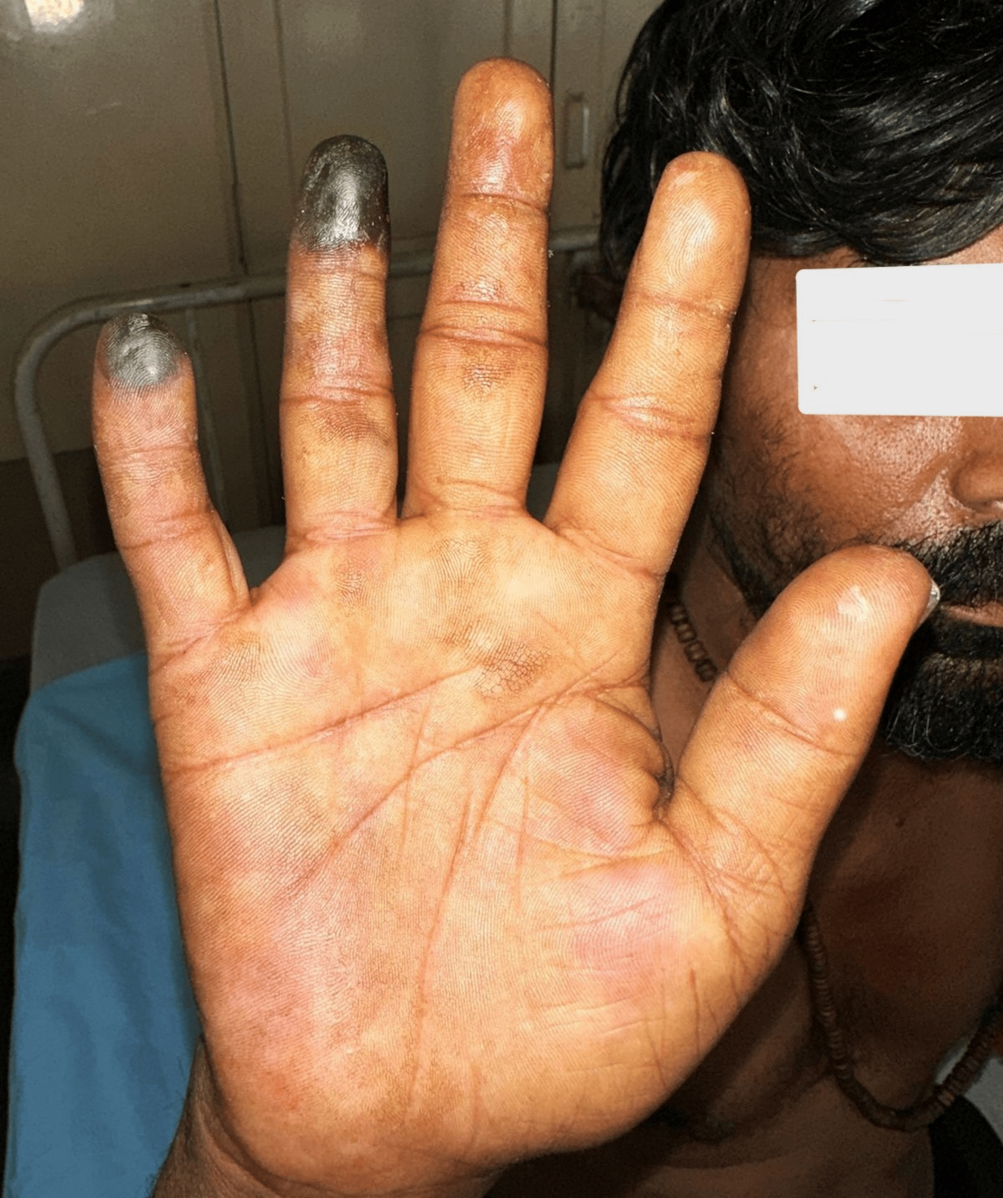
Post-imatinib therapy photograph showing demarcation in the ring and little fingers with an improvement of pre-gangrenous changes in the index and middle fingers

Our patient responded very well to chemotherapy clinically with the normalization of pre-gangrenous changes in the index and middle fingers and hematological reduction in platelet count to 2.2 lakhs after three weeks of therapy initiation (Figure 4). Complete hematological response was noted at the third month of treatment initiation. The tip of the ring and little fingers with dry gangrene with a clear line of demarcation were allowed to go for autoamputation as the patient was not willing for surgery. The patient is still in the follow-up period.

## Discussion

A myeloproliferative neoplasm called CML contains aberrant clonal hyperproliferation of myeloid cells in the bone marrow. It can manifest clinically as an acute blast phase to a more indolent chronic phase. Easy fatigability, anemia, recurrent infections, appetite loss, and splenomegaly characterize the chronic phase of CML. Leukocytosis, thrombocytosis, and neutrophilia are among the hematological findings. The BCR/ABL translocation gene, also discovered in our patient, is typically used to confirm the conclusive diagnosis of CML [1].

CML with primary symptoms of digital gangrene is an infrequent entity. That ischemic symptoms like digital gangrene further asserted it is never found as the primary clinical manifestation of CML [1,2]. Thakral et al. reported such a rare presentation, where the patient had digital gangrene secondary to CML with thrombocytosis similar to our case report detected incidentally while evaluating the cause of digital gangrene.

Cancer-associated digital ischemia may antedate, coincide, or follow the diagnosis of cancer, or it can signal the disease's recurrence [3]. According to Poszepczynska-Guigné et al., cancer-related digital ischemia often coincides with (47%), precedes (44%), or follows (9%) a cancer diagnosis. Digital ischemia associated with cancer is most commonly seen in adenocarcinomas (41%), hematological malignancies (19%), and CML in one case. In our situation, acral vascular symptoms appeared before CML was identified.

To date, the exact etiology behind digital gangrene in CML remains unclear, but many hypothetical factors have been put forward, such as local factors like abnormal clonal cell-endothelium interaction, arteritis brought on by the deposition of tumor antigen-antibody complexes or as a result of immune dysregulation, blood hyperviscosity causing sluggish blood flow (due to circulating blast cells), and hypercoagulability due to circulating coagulant factors (cancer procoagulant microparticles, tumor mucin), impaired anticoagulant and fibrinolytic pathway, peripheral thrombosis, or drug toxicity [2,3,5,6]. In our case, we could exclude thrombosis by normal arterial Doppler of the upper limb and autoimmune arteritis by the absence of antinuclear antibodies and antiphospholipid antibodies. In myeloproliferative diseases, severe thrombocytosis leading to thrombotic consequences is a common finding [6]. In contrast to myeloproliferative diseases, CML rarely exhibits symptomatic thrombocytosis [6]. Additionally, our individual had CML with moderate thrombocytosis, but we could not establish a causal link between it and digital gangrene. Only three case reports of digital ischemia due to thrombocytosis in patients with CML [1,2,6] have been described in the literature. As far as we know, this is the fourth case report of CML-related digital ischemia with gangrene. Initial treatments for patients in all three case studies included the use of hydroxyurea [1] or hydroxyurea combined with aspirin and allopurinol [2-6]. In light of the patient's persistent symptomatology and nonresponse to medical therapy with hydroxyurea, plateletpheresis was discovered to be an efficient treatment option with promising outcomes [1,2,6]. Thrombocytosis is thus identified as a significant factor for digital gangrene in CML [1,2,6]. However, in our situation, the patient did not respond to hydroxyurea or low-molecular-weight heparin at first but did respond favorably to the tyrosine kinase inhibitor imatinib and aspirin regarding clinical and hematopoietic response. Within three weeks of imatinib therapy initiation, the patient showed complete remission of pre-gangrenous changes in the index and middle digits, along with a decrease in platelet count. By the third month of treatment, the patient's leukocyte count had returned to normal, consistent with a case report by Byun et al. [7]. It is unclear exactly how thrombocytosis manifests as symptoms. However, it may be related to the increased viscosity of blood due to thromboxane-mediated aggregation and sludging of the platelet mechanism. Therefore, aspirin is advised as the first line of treatment for essential thrombocytosis [9].

Imatinib therapy has shown excellent results in patients with chronic-phase CML, with complete cytogenetic remission seen in 76.2% and improvement in long-term survival in 89% [7,9]. We also saw positive clinical and hematological responses throughout a three-month follow-up period. Imatinib is now licensed for use as the medicine of preference in the treatment of CML patients who are in the chronic phase [9].

## Conclusions

CML presenting with digital gangrene as a primary symptom is extremely rare. Clinicians should consider the possibility of underlying malignancy as a rare cause of digital gangrene with rapid onset. Hyperviscosity and leukostasis are the probable causes of digital gangrene in CML. Early diagnosis and chemotherapy treatment will improve the patient's prognosis. Currently, imatinib is the drug of choice in managing CML in the chronic phase.
